# Involving Undergraduates in Publishable International Research: Experiences in Latin America

**DOI:** 10.3389/fpsyg.2019.00656

**Published:** 2019-03-29

**Authors:** Brien K. Ashdown

**Affiliations:** Department of Psychology, Hobart and William Smith Colleges, Geneva, NY, United States

**Keywords:** Latin America, undergraduate research, international research, guatemala, cultural psychology

Involving undergraduates in research is important to many sciences such as biology, chemistry, physics, and psychology (Russell et al., 2007; Thiry and Laursen, 2011). Cultural psychological research often occurs in “the field,” far from home. This scholarship has specific challenges that can make it difficult to involve undergraduates. These challenges, however, are worth the rewards of introducing them to international and culturally-based research. Here, I provide suggestions from my experiences guiding 26 undergraduates while conducting psychological research in Guatemala. This work has resulted in five publications (including nine undergraduate co-authors), various conference presentations, and other ongoing projects with another seven student collaborators (García Egan et al., 2014; Faherty et al., 2016; Ashdown and Buck, 2018; Ashdown et al., 2018; Rohner et al., 2019).

Many challenges of conducting research with undergraduates while abroad are similar to working with undergraduates on campus. Undergraduates are research novices, requiring significant supervision and training (Shellito et al., 2001; Thiry and Laursen, 2011). Challenges specific to international research, like working in unfamiliar locations and avoiding specific cultural and ethical pitfalls, can be managed by focusing on five issues: (1) establish local collaborations, (2) avoid “safari” research, (3) understand students' cultural and research skills, (4) get official institutional support for students' travel and work, and (5) model international research ethics.

## Establish Local Collaborations

International research requires partnerships with local collaborators (Pao, 1992; Ashdown and Buck, 2018). Such collaborations are more successful (Pao, 1992), and local collaborators have better access to local populations, understand local customs, and can serve as cultural ambassadors in addition to collaborators. It is important to model these collaborations for undergraduates to teach them the value of such collaborators. Working with local collaborators can also lead to the opportunity to include local undergraduates as collaborators, a worthy goal of any international psychologist. For example, I worked with two Guatemalan students on a project exploring Guatemalan mothers' parenting beliefs (García Egan et al., 2014). I was introduced to these student collaborators through my previous collaborations with other local Guatemalan scholars.

Working with local collaborators helps researchers avoid falling into the trap of the “White savior complex” (Straubhaar, 2014; Belcher, 2016; Bex and Craps, 2016; Jailani, 2016; Ashdown and Buck, 2018). This complex occurs when researchers (usually highly-educated, relatively wealthy White people from the Global North) view themselves (and are sometimes viewed by participants) as having all necessary skills and knowledge to research an issue or solve a problem. With deep roots in colonialism (Rigney, 1999; Rios, 2015; Aronson, 2017; Ashdown and Buck, 2018), this behavior should be avoided at all costs. Conducting research in partnership with local collaborators ensures investigators avoid culturally imperialistic research practices (Dupre, 1994; Wilmshurst, 1997), while teaching undergraduates this important practice.

## Avoid “Safari” Research

The term “safari research” describes scholarship by researchers who lack deep understandings of the cultures they study. This practice is unethical, promotes the White savior complex, and should never be modeled for undergraduates. Otherwise, scholars may reify the structures of colonialism in their work and perpetuate it in a new generation of researchers. Instead, researchers should limit themselves to working in cultural contexts where they have experience, and where they can receive support from, and in turn support, local collaborators. This issue is closely tied the White savior complex, and of such importance that I recently published a critique of the way cultural psychologists interact with foreign cultures (Ashdown and Buck, 2018). This publication has an undergraduate author and is based on her honors thesis—a good example of how students can do good scholarship *about* cultural psychology as well as *within* cultural psychology.

Safari research intensifies the complexities of working in a foreign language. I believe researchers should not work in a language they cannot speak. Even when fluent in a second language, they should work with local collaborators who are native speakers. Translations of measures and surveys should always utilize rigorous back-translation processes that involve native speakers of the language—regardless of how well a researcher might speak both languages (Brislin, 1970; Dorcas et al., 2000; Hambleton and Zenisky, 2011).

Language is an area where undergraduate students can be of great help. In past projects, I involved undergraduates who were native Spanish speakers (often bilingual in Spanish and English) to help with the translation process. While translation work alone does not meet the requirements for authorship, it is valuable and always recognized in the authors' note of publications and presentations (Ashdown et al., 2017). Often, this effort serves as the first step, or “try out” aspect, of getting a new undergraduate researcher involved in my scholarship.

Related to the complexities of language is an awareness of local cultural norms (Rigney, 1999; Rogler, 1999; Finnemore, 2009). It is impossible for safari researchers to practice ethical scholarship in a culture where they do not understand customs surrounding concepts like gender relations or social hierarchies. When I take undergraduates abroad, I require a significant amount of reading and meetings before departure. These are not replacements for the years of immersion necessary for cultural proficiency (Ruben, 1989), but serve to prepare undergraduates for international field work. For example, students who work with me read *The Guatemalan Reader* (Grandin et al., 2011) before our trip and meet with me 2–3 times a month to discuss their reading.

Finally, for students (or other researchers) hoping to begin a research program in a culture with which they are currently unfamiliar there are some tactics that will help them avoid the pitfalls of safari research. Begin establishing relationships with potential local collaborators before traveling (e.g., via email listservs and Internet groups), and try to build bridges with other international researchers working in the same area with an eye toward future collaborations and a “foot in the door” to learn about the culture. And, in the end, it is better to take someone with you who is familiar with the culture (or hire a translator to accompany you) than it is to become a safari researcher.

## Understand and Support Students' Current Skills

Many undergraduates have international experience, and may have studied abroad in cultures similar to the one where you work. They come with valuable cultural skills and understanding—though their experience maybe more superficial than you would like. It is important to talk with these students to determine the intercultural skills they have developed from their experiences and what others need cultivating.

Students without international experience can still be valuable research assistants. They do not need to travel to conduct research, nor do they need previous international experiences to be important assets in international scholarship. Students who do not travel to collect data can be members of a research team assigned other tasks. On my team, these students aid in data analysis, literature searches, and writing. In all of my publications based on data from Guatemala that have undergraduate co-authors, some of those authors did not travel with me, but did significant work on writing once I returned to campus with data. I still require them to have a solid grounding in Guatemalan culture—just as students who travel with me are required to complete certain readings and meet to discuss cultural and current events, so are students who do not.

Keep in mind that whether or not students have relevant cultural experience, it does not compensate for a lack of research skills. Undergraduates conducting international research need support and training in basic skills related to the project. These skills often include interviewing, managing focus groups, or navigating local research customs. The line between cultural skills and research skills can blur—a student with great interviewing skills still needs to understand local norms around social interactions and speak the language in which the interviews occur. Working with undergraduates in international contexts requires that you balance the need for these skills with providing the students the experiences needed to gain and develop the skills.

## Obtain Institutional Support

Institutional support is important for any scholarship involving undergraduates; this support is particularly important for international research, as travel costs can exceed what many researchers can pay, and there can be higher liability connected to travel. Because of the costs of traveling, my institution has a few competitive scholarships to support students' international work. Official institutional support may also make it easier for students to apply for external funding.

Keep in mind that while having institutional support makes the process of traveling with students to conduct research more feasible for various reasons, it may not be necessary (you should check institutional policies). I have traveled with students both as part of an official program with my institution and as individual students (or small groups of students) accompanying me during summer break. The comfort you have traveling without official institutional support should have the largest influence on how you make this decision.

One aspect of this decision might be whether the institution will provide liability protection for you, which is something I always consider when I take students to Guatemala. I have yet to experience personal risk while traveling with students, but I take steps to protect myself. For example, I edited a copy of the legal liability paperwork students complete, sign, and notarize before they travel on an official institutional program (e.g., study abroad) so that it acts as a contract between me and the students. It serves the purpose of protecting me from liability in many situations. I also suggest having a discussion with your insurance agent about possible insurance coverage.

## Model Good International Research Ethics

My last suggestion, to model good research ethics, may seem like a suggestion that all researchers should follow. I believe this modeling deserves special consideration when conducting international research. As mentioned previously, these ethics include avoiding a “White savior complex,” getting nowhere near cultural or scientific colonialism, and not engaging in safari research. It also includes a few methodological ethics specific to international research.

First, we should always get Institutional Review Board (IRB) approval for any research that involves human participants (or an Institutional Animal Care and Use Committee for non-human participants), which clearly is not specific to international research (Amdur and Biddle, 1997; Oakes, 2002). This, however, is not enough. We should ensure that we get approval from a local IRB, too (Greene and Geiger, 2006; Ravina et al., 2009). If there is not an appropriate and relevant local IRB, we should get ethics approval from an authorized and appropriate person in the community or organization where we are working, such as a village elder, elected official, or program director.

Second, in addition to working with local collaborators, we should ensure that collaborators receive appropriate authorship recognition. No matter where our collaborators live and work, if their effort on our project would constitute authorship recognition on a publication or presentation for a USA-based colleague, our international collaborators are entitled to that same recognition. Not including collaborators as authors simply because they do not work in a traditional research setting such as university (e.g., community organizers, program directors, etc.,) is inappropriate. Simply put, all contributions to a project should be ethically and appropriately recognized.

Third, and related to the second point, I have made a conscious decision to publish my Guatemala-based work in journals that are accessible to local Guatemalan scholars. This decision often means publishing in open-access journals because many Guatemalan scholars cannot afford the excessive cost of accessing databases and journals. Because many highly-ranked open-access journals have hefty publication fees (which my institution will not pay), I often choose to publish in good journals that are not at the top of the journal ranks. Otherwise, I would find it difficult to ethically justify my research because it would not be accessible to my collaborators, their institutions, or other local scholars (Kansa et al., 2013; Butler, 2016; Schiltz, 2018). For example, I have published with student collaborators in the *Revista Interamericana de Psicolog*í*a* (García Egan et al., 2014), the *Psi Chi Journal of Psychological Research* (Faherty et al., 2016), and the *Acta de Investigación Psicológica* (with a recently graduated local collaborator; Gomez and Ashdown, 2013).

## Conclusion

Involving undergraduates in high-quality international research is one of the aspects of my career I most enjoy. As a cultural psychologist, my research occurs in the context of the beautiful, colorful, and exciting culture and geography of Guatemala. While working with students is a highlight of my work, it can be challenging to involve undergraduates in this type of research process. I have found, though, that challenges pale in comparison to the rewards that come from introducing my undergraduates to the process of international and culturally-based research, and to the splendor of Guatemala.

## Author Contributions

The author confirms being the sole contributor of this work and has approved it for publication.

### Conflict of Interest Statement

The author declares that the research was conducted in the absence of any commercial or financial relationships that could be construed as a potential conflict of interest.
